# Changes in Serum Cytokines May Predict Therapeutic Efficacy of Tofacitinib in Rheumatoid Arthritis

**DOI:** 10.1155/2019/5617431

**Published:** 2019-10-24

**Authors:** Yuxuan Li, Lin Yuan, Jie Yang, Yue Lei, Hui Zhang, Liping Xia, Hui Shen, Jing Lu

**Affiliations:** Department of Rheumatology and Immunology, The First Affiliated Hospital of China Medical University, 155 Nanjing North Street, Shenyang, Heping District 110001, China

## Abstract

**Objective:**

Tofacitinib is a novel therapy for rheumatoid arthritis (RA). The aim of this study was to measure various serum cytokines levels and to explore potential markers predictive of therapeutic efficacy of tofacitinib for RA patients.

**Methods:**

Thirty-two patients with RA were given tofacitinib (5 mg bid). Serum cytokines levels of Th1 (IFN-*γ*), Th2 (IL-6), Th17 (IL-17), Tregs (IL-35), and TNF-*α* were detected by enzyme-linked immunosorbent assays.

**Results:**

Disease activity was significantly decreased as early as week 4 after tofacitinib treatment. Serum IL-35 levels were significantly increased and serum levels of TNF-*α*, IL-17, IL-6, and IFN-*γ* were significantly reduced in response to tofacitinib since week 4.

**Conclusions:**

After treatment with tofacitinib, RA patients may benefit from monitoring of disease activity as early as week 4. IL-35 also might be a predictive indicator of the disease activity and drug efficacy. Meanwhile, tofacitinib might be CS-sparing in RA.

## 1. Introduction

Rheumatoid arthritis (RA) is a chronic autoimmune disease characterized by inflammatory cartilage, joint, and bone destruction [1]. Significant developments in clinical outcomes have been accomplished over the past decades with the adoption of earlier and targeted medication including maximizing the utility of conventional synthetic disease-modifying antirheumatic drugs (csDMARDs), biologic disease-modifying antirheumatic drugs (bDMARDs), and the introduction of treat-to-target approach. Furthermore, it is currently strongly recommended that corticosteroid (CS) could be used in combination with DMARDs to wait for the response of DMARDs and then to taper the CS dosage as soon as possible [2]. In China, nearly 85% of patients with RA have used or are using bDMARDs or csDMARDs in their clinical practice, but less than 30% of patients have achieved clinical remission or low disease activity [3], suggesting an unmet need for additional novel therapies.

Tofacitinib is the first Janus kinase (JAK) inhibitor which is approved for the treatment of RA. Proinflammatory cytokine activation of the JAK/signal transducers and activators of transcription (STAT) signal transduction pathway is a key event in the pathogenesis of RA. Cytokines such as interleukin- (IL-)6, interferon- (IFN-)*α* triggers receptor-related JAKs through binding to its intracellular receptors, which act as docking sites for STAT [4]. Pharmacologically, tofacitinib specifically blocks signaling by cytokine receptors which relate to JAK1 and/or JAK3 [5]. It has also been demonstrated that changes of the serum cytokines may be a crucial action of tofacitinib for RA. However, alterations in various serum cytokines prior to and after tofacitinib treatment have not been extensively explored.

In the current study, we explored patients with RA who were treated with tofacitinib. We measured serial changes in serum cytokines to investigate the effect of tofacitinib treatment on patients with RA over a 24-week period and to identify serum markers which could have relevance to disease activity, antibody production, or efficacy of tofacitinib.

## 2. Materials and methods

### 2.1. Patients

A total of 32 patients with RA at the First Affiliated Hospital of China Medical University were recruited for this study. The recruited patients all met the American College of Rheumatology criteria for RA [6]. None of the patients had undergone biological treatment (i.e., infliximab or adalimumab). These patients were recommended that in preference be given tofacitinib in combination with csDMARDs. Tofacitinib was administered at 5 mg twice a day. Serum samples were stored from 0, 4, 8, 12, 16, to 24 weeks of medication. Blood tests for erythrocyte sedimentation rate (ESR) and C-reactive protein (CRP) were measured by Westergren method, immune transmission turbidity method, respectively. Rheumatoid factor (RF) and anticyclic citrullinated peptide antibodies (ACPAs) were assessed by immunoturbidimetric assays and chemiluminescence analysis, respectively. Each patient's swollen joint count (SJC) and tender joint count (TJC) using were assessed 28-joint count. Patient- and physician-reported outcomes are as follows: pain visual analog scale (VAS) (0–10 scale) and Health Assessment Questionnaire-Disability Index (HAQ-DI) (0–3 score; high values indicate reduced physical function). Written informed consent was provided by all subjects, and the study was approved by the ethics committee of the First Affiliated Hospital of China Medical University and was conducted according to the Declaration of Helsinki.

### 2.2. Assessment of Disease Activity and Efficacy of Tofacitinib

The DAS28-ESR was used for the determination of disease activity at 0, 4, 8, 12, 16, and 24 weeks after tofacitinib treatment [7]. Clinical response was defined as reaching low disease activity according to DAS28 ≤ 3.2 for 6 months or DAS28 remission (DAS28 < 2.6) for 6 months [8, 9]. Response to tofacitinib treatment was evaluated at 0, 4, 8, 12, 16, and 24 weeks by DAS28-ESR. The responder was defined as a patient with a clinical response (DAS28 ≤ 3.2) at week 24 after initiation of tofacitinib treatment, while the nonresponder was defined as a patient without clinical response (DAS28 > 3.2) at week 24 after initiation of the tofacitinib treatment.

### 2.3. Measurement of Serum Cytokines Levels

The serum levels of cytokines including IFN-*γ* (type 1 T-helper cells (Th1)), IL-6 (type 2 T-helper cells (Th2)), IL-17 (type 17 T-helper cells (Th17)), IL-35 (regular T cells (Tregs)), and tumor necrosis factor- (TNF-)*α* were measured at weeks 0, 4, 8, 12, 16, and 24 using commercially enzyme-linked immunosorbent assay (ELISA) kit (IL-17, IL-6, TNF-*α*, and IFN-*γ*: R&D Systems Minneapolis, MN, USA; IL-35: Shanghai ShuangYing, China) according to the manufacturer's protocols. Each sample was tested in duplicate. The optical density was measured at 450 nm using an automatic ELISA reader.

### 2.4. Statistical Analysis

Continuous variables with normal distribution were presented as mean ± standard deviation (SD); nonnormal variables were presented as median (interquartile range (IQR)). Student's *t*-test was used for analysis of continuous variables between two groups. Spearman's correlation coefficient was used to explore the associations between two variables. All analyses were performed using SPSS17.0 (SPSS, Inc., Chicago, IL) and GraphPad Prism 6 software. Differences of *P* < 0.05 were considered significant.

## 3. Results

### 3.1. Patients

The RA patients had a median (IQR) age of 44 (31–59) years. The disease duration with a median (IQR) was 6.1 (5.2–15.0) years. Before starting treatment, 71.9% of RA patients in this study had high disease activity. Among these 32 patients, 31 (96.9%) were RF positive and 23 (71.9%) were ACPA positive. All patients with RA were taking tofacitinib with concomitant csDMARDs including methotrexate, leflunomide, sulfasalazine, and hydroxychloroquine. CS (prednisone equivalent 5–10 mg/day) was used in 8 patients with RA to help to control disease activity (Table 1).

### 3.2. Efficacy and Safety of Tofacitinib

**Figure 1:**
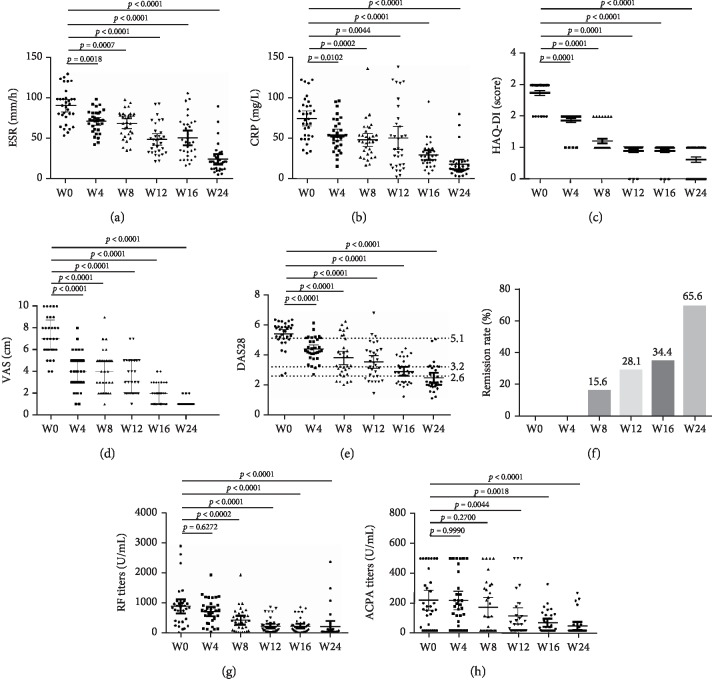
Changes in ESR (a), CRP (b), HAQ-DI (c), VAS (d), DAS28 (e), RF (g), and ACPA (h) in patients treated with tofacitinib. In (f), the dotted horizontal lines indicate borders between categories of DAS28: (i) high (DAS28 > 5.1); (ii) moderate (3.2 < DAS28 ≤ 5.1); (iii) low (2.6 ≤ DAS28 ≤ 3.2); and (iv) patients in remission (DAS28 < 2.6). The *x*-axis has six measurement time points indicated as 0, 4, 8, 12, 16, and 24 weeks after tofacitinib treatment. Abbreviations: ESR: erythrocyte sedimentation rate; CRP: C-reaction protein; HAQ-DI: Health Assessment Questionnaire-Disability Index; VAS: visual analogue scale; DAS28: disease activity score in 28 joints based on erythrocyte sedimentation rate; RF: rheumatoid factor; ACPAs: anticyclic citrullinated peptide antibodies.

After tofacitinib treatment was initiated, ESR, CRP, HAQ-DI score, pain VAS score, and DAS28 were significantly decreased as early as week 4 (*p* = 0.0018, *p* = 0.0102, *p* < 0.0001, and *p* < 0.0001, respectively) (Figures 1(a)–1(d)). Most RA patients were in the high disease activity before tofacitinib treatment and was started with a mean (IQR) DAS28 of 5.6 (5.0-5.9) (Figure 1(e)). After the onset of tofacitinib treatment, most RA patients moved to moderate and low disease activity (*p* < 0.0001) since week 8. At week 24, the rate of remission in RA patients reached 65.6% (Figure 1(f)). Furthermore, RF titers and ACPA titers were significantly decreased at week 8 and week 12, respectively, as well (*p* < 0.0002 and *p* = 0.0044, respectively) (Figures 1(g) and 1(h)). In terms of tofacitinib safety, there were no obvious abnormalities in hematologic and hepatic system, renal function including hemoglobin, white blood cell count, platelet, serum aspartate aminotransferase, alanine aminotransferase, creatinine, and blood urea nitrogen. No cases of herpes virus infection were reported. However, 2 cases suffered upper respiratory tract infections at week 8 and week 12, respectively.

### 3.3. Changes in Serum Levels of Cytokines in Response to Tofacitinib

**Figure 2:**
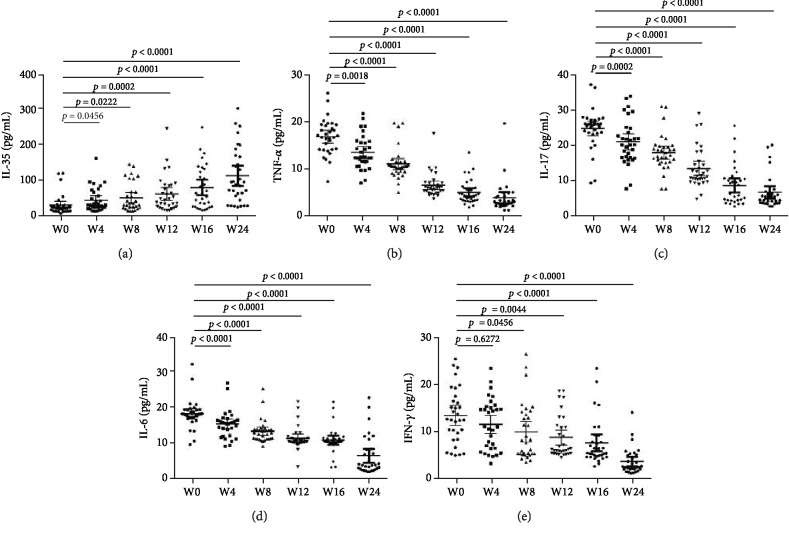
The serum cytokines levels in the RA patients after tofacitinib treatment at the week 0, 4, 8, 12, 16, and 24. (a) IL-35; (b) TNF-*α* (c) IL-17; (d) IL-6; (e) IFN-*γ*. Abbreviations: IL: interleukin; TNF: tumor necrosis factor; IFN: interferon. The *x*-axis is the six measurement time points indicated as 0, 4, 8, 12, 16, and 24 weeks after tofacitinib treatment is initiated.

The median (IQR) at baseline serum levels of cytokines is shown in Table 1. After tofacitinib treatment, serum levels of TNF-*α*, IL-17, IL-6, and IFN-*γ* significantly decreased and serum levels of IL-35 significantly increased during the observation period (Figure 2).

### 3.4. Baseline Serum Levels of Cytokines

We explored the associations of serum levels of cytokines at baseline and clinical parameters. The results are summarized in Table 2. It is noteworthy that baseline serum IL-35 levels (week 0) were negatively correlated with inflammatory parameters: ESR and CRP (*r* = −0.4, *p* = 0.0300; *r* = −0.5, *p* = 0.0013, respectively). This association was consistent with the changes in HAQ-DI score, VAS score, and DAS28, respectively (*r* = −0.4, *p* = 0.0202; *r* = −0.4, *p* = 0.0164; *r* = −0.4, *p* = 0.0100, respectively). However, for autoantibody production, no significant associations were found between serum IL-35 levels and RF and ACPA titers in RA patients (*r* = 0.1, *p* = 0.6721; *r* = 0.1, *p* = 0.6037, respectively). These associations were similar with those between TNF-*α* and IL-6.

### 3.5. Comparison of Responders and Nonresponders at Week 24 after Initiation of Tofacitinib

At week 24, 25 of the 32 patients (78.1%) were responders and 7 patients (21.9%) were nonresponders.  Table 3 compares the clinical parameters and serum cytokines levels between responders and nonresponders at week 24 after initiation of tofacitinib, respectively. We found that prior to treatment, at week 24, serum levels of IFN-*γ*, IL-6, and IL-17 were significantly lower in responders than in nonresponders (*p* < 0.0001, *p* = 0.0113, and *p* = 0.0048, respectively). Serum levels of IL-35 were significantly higher in responders than in nonresponders (*p* = 0.0011). However, serum levels of TNF-*α* at week 24 did not differ between responders and nonresponders (*p* = 0.0943).

### 3.6. Change of the Mean Daily CS Dose (Prednisone Equivalent)

**Figure 3:**
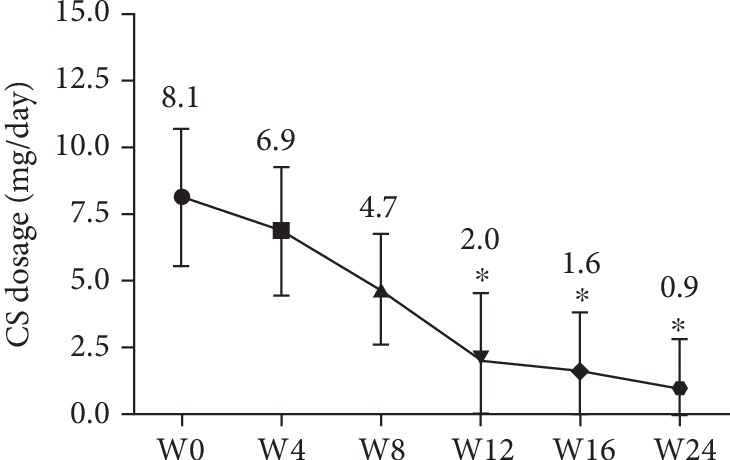
Change of the mean daily CS dose (prednisone equivalent) during the 24 weeks after tofacitinib treatment is initiated. Abbreviations: CS: corticosteroid. ^∗^*p* < 0.05 versus CS dosage at baseline (week 0). Data was represented as mean ± SD. ^∗^*p* < 0.05 versus week 0.

The study also observed the CS sparing after initiation of tofacitinib. At the initiation of tofacitinib, 25% of patients (*n* = 8) were administrated with a daily dose of 5 mg or 10 mg of CS. During the study period, there was a significant sparing effect of CS as early as week 4. The mean daily dose of oral CS fell from 8.1 mg at baseline to 6.9 mg at week 4, 4.7 mg at week 8, 2.0 mg at week 12, 1.6 mg at week 16, and 0.9 mg at week 24 (Figure 3). Besides, 25.0% of patients (*n* = 2) were treated with a daily dose of 5 mg of CS at week 4 and 50.0% of the patients (*n* = 4) were able to stop CS therapy at week 12. At the end of observation period, 6 patients had stopped CS therapy, the remaining 2 patients tapered their CS dose to ≤5 mg/day.

## 4. Discussion

2016 European League Against Rheumatism recommendations for the management of RA stated that tofacitinib could be considered a first-line molecular-targeted therapy [10]. Previous data on phase II and phase III trials showed that the tofacitinib was useful in attenuating symptoms of RA in biologic-naïve patients and patients who had failed previous treatment with biological agents [11–14]. Tofacitinib and MTX combination therapy is effective in the treatment of RA with an inadequate response to MTX. In current study, ESR, CRP, HAQ-DI score, pain VAS score, and DAS28 all significantly decreased at week 4 indicating tofacitinib was indeed effective, and these parameters as early as week 4 can predict efficacy in response to tofacitinib treatment. Given the current and previous data, tofacitinib provided an efficacious treatment choice for patients with RA and could be timely monitored of efficacy as early as 4 weeks.

The pathogenesis of RA is based on multiple cytokines regulated by T cell-activated signaling pathways, which can promote communication between immune cells and are effective as markers for immune response. CD4+Th lymphocytes can be divided into Th1 (IFN-*γ*, IL-2, and TNF-*β*) and Th2 (IL-4, IL-6, IL-10, and IL-13) subsets on the basis of their cytokines secretion profile [15]. In addition to well-characterized Th1 and Th2 lymphocytes, naïve CD4+T cells can also be differentiated into Th17, a distinct subset of Th cells characterized by expression of IL-17. Another subpopulation of CD4+ lymphocytes is Tregs. It included IL-10, TGF-*β*, and a novel cytokine IL-35 and could refrained autoreactive effector T cells. In this study, we measured the serum levels of a range of cytokines (IL-35, IL-17, IL-6, TNF-*α*, and IFN-*γ*); these cytokines represented a common range of functional immune messengers.

During the course of the tofacitinib treatment, serum TNF-*α*, IL-17, IL-6, and IFN-*γ* were significantly decreased. Serum IL-6 and IFN-*γ* levels were rapidly reduced throughout the therapy could best be understood as a reaction to targeted JAK blockage [16]. Although tofacitinib theoretically did not directly inhibit the production of TNF-*α* and IL-17, their levels were also downregulated during the observation period. There may be a synergistic crosstalk between TNF-*α*, IL-17, and IL-6.

IL-35, firstly identified in 2007, is a novel member of the IL-12 family which included IL-12, IL-23, and IL-27 [17]. In addition, IL-35 is mainly produced by Tregs, triggering its anti-inflammatory effect in RA [18–20]. IL-35 has been demonstrated to play an important role in pathogenesis of RA [21, 22]. Our previous study also showed that IL-35 inhibits angiogenesis and inflammation in collagen-induced arthritis-derived fibroblast-like synoviocytes [23]. Furthermore, as noted in review articles, IL-35 appears to exhibit immunosuppressive properties in other autoimmune diseases including systemic lupus erythematosus, systemic sclerosis, psoriatic arthritis, Sjögren syndrome, and idiopathic inflammatory myopathies [24], which suggest that progress in the IL-35 field of research in autoimmune diseases is promising. In our study, during the course of tofacitinib treatment, the median (IQR) levels of IL-35 at week 24 were higher than those at week 0. These changes could be explained by inflammatory Tregs, which was the main source of IL-35, which have expanded after initiation of tofacitinib. To better understand the role of IL-35 in RA, we tried to figure out the associations between IL-35 and clinical parameters. In our study, serum IL-35 levels at baseline were negatively correlated with ESR, CRP, HAQ-DI score, pain VAS score, and DAS28. This finding coincided with experimental results of Nakano et al. [21]. These observations indicated that the IL-35 could be useful to assess disease activity of RA, further suggesting that IL-35 is closely related to the immune response of RA patients. However, the definite mechanism about how IL-35 works in RA warrants further investigation. Additionally, Table 2 also shows that the baseline levels of IL-6 and TNF-*α* were also positively correlated with disease activity respectively, which were coincident with previous studies [25–28].

Meanwhile, RF and ACPA titers were decreased at week 8 and week 12, respectively, in response to tofacitinib. In our study, we did not show the relationship between IL-35 and autoantibody, including RF and ACPA. The underlying speculation for that may be some reasons. First, RF and ACPA have been applied to the diagnosis of RA, but neither provides enough sensitivity and specificity. Second, the study lacks molecular mechanisms underlying the involvement of IL-35 in the pathogenesis of RA. More studies from in vivo experimental models are necessary to inspect the special role of IL-35 in the development of RA.

We also investigated whether the production of any of the 5 cytokines differed before or 24 weeks after tofacitinib treatment between responders and nonresponders. In these nonresponders, 6 of 7 patients (85.8%) were female. However, in responders, 19 of 25 (76.0%) were female. It is worth noting that the *p* value between the sex ratio of the two groups was 0.69, suggesting that the prevalence of female in nonresponders seemed to be insignificant and should be validated in future studies with a larger number of patients.

In our study, tofacitinib led to significant CS sparing as early as 3 months of RA treatment. The study showed a significant decrease of 47.0% of CS dose in the 6 months of treatment with tofacitinib. Among patients on CS at tofacitinib onset, CS dose was decreased in nearly half. CS therapy in RA has anti-inflammatory effect and is efficacious for short-term symptomatic relief. However, such therapy has many side effects such as bone loss and infection. Therefore, it is strongly recommended that the CS dosage be maintained at a minimum dosage and to attempt to reduce CS dosage in the case of low disease activity or remission [29]. CS sparing seems to be a standard for adjusting DMARDs therapy, especially when the dose of CS is ≥10.0 mg/day [30]. Taken together, in patients with RA, tofacitinib may have a CS sparing effect which will assist physicians to reduce CS dosage in real life practice as early as the first 12 weeks of treatment.

Roy Fleischmann and his colleagues have reported that the most common adverse permanent discontinuation events were elevated alanine aminotransferase (ALT) (0-2.9%) and elevated aspartate aminotransferase (AST) (0-2.9%) and serious infection events including pneumonia, herpes zoster, pyelonephritis, pneumococcal sepsis, ankle-joint infection, respiratory tract infection, urinary tract infection, infectious gastroenteritis, and the exacerbation of Whipple's disease that occurred at a rate of 0-5.4% [14, 31]. In this study, there were no obvious abnormalities in hematologic and hepatic system, renal function including hemoglobin, white blood cell count, platelet, serum ALT, AST, creatinine, and blood urea nitrogen. However, 2 cases suffered upper respiratory tract infections at week 8 and week 12, respectively. No cases of other infections were reported.

The current study has some limitations. The sample studied was small, which might result in a type II error during the comparison. Therefore, our data need to be confirmed for the clinical utility of the identified cytokines. However, we believe that this study is necessary because this is the first study determining serum cytokines including Th1 (IFN-*γ*), Th2 (IL-6), Th17 (IL-17), Tregs (IL-35), and TNF-*α* in RA patients treated by tofacitinib.

## 5. Conclusions

In conclusion, patients with RA may benefit from monitoring of disease activity as early as week 4 after tofacitinib treatment. Tofacitinib increased serum levels of IL-35 and decreased serum levels of IFN-*γ*, IL-6, IL-17, and TNF-*α*. IL-35 might be an indicator of the disease activity and tofacitinib efficacy. Tofacitinib might be CS sparing in RA.

## Figures and Tables

**Table 1:** Baseline characteristics of patients with RA.

Characteristics	Patients with RA (*n* = 32)
*Demographics*	
Age, median (IQR), years	44 (31-59)
Sex (F/M)	25/7
Disease duration, median (IQR), years	6.1 (5.2-15.0)
*Disease characteristics*	
ESR, median (IQR), mm/h	91.0 (76.6-98.8)
CRP, median (IQR), mg/L	73.3 (52.8-90.7)
TJC, median (IQR)	7 (2-23)
SJC, median (IQR)	7 (1-20)
HAQ-DI, median (IQR)	2.7 (2.2-3.0)
Pain VAS, median (IQR), cm	7.0 (6.0-8.8)
DAS28, median (IQR)	5.6 (5.0-5.9)
RF positive, *n* (%)	31 (96.9%)
ACPA positive, *n* (%)	23 (71.9%)
*Concomitant medications*	
MTX, *n* (%)	15 (46.9)
LEF, *n* (%)	13 (40.6)
SSZ, *n* (%)	4 (12.5)
HCQ, *n* (%)	23 (71.9)
NSAIDs, *n* (%)	26 (81.3)
CS, *n* (%)	8 (25.0)
Oral prednisone dose (mg/day)	10 (5-10)
*Baseline serum levels of cytokines*	
IFN-*γ*, median (IQR), pg/mL	12.5 (8.6-17.2)
IL-6, median (IQR), pg/mL	18.2 (17.2-19.4)
IL-17, median (IQR), pg/mL	25.5 (23.1-26.9)
IL-35, median (IQR), pg/mL	22.4 (15.1-28.0)
TNF-*α*, median (IQR), pg/mL	16.7 (14.0-19.2)

Abbreviations: RA: rheumatoid arthritis; IQR: interquartile range; F: female; M: male; ESR: erythrocyte sedimentation rate; CRP: C-reaction protein; TJC: tender joint count; SJC: swollen joint count; HAQ-DI: Health Assessment Questionnaire-Disability Index; VAS: visual analogue scale; DAS28: disease activity score in 28 joints based on erythrocyte sedimentation rate; RF: rheumatoid factor; ACPAs: anticyclic citrullinated peptide antibodies; NSAID: nonsteroidal anti-inflammatory drug; CS: corticosteroid; MTX: methotrexate; LEF: leflunomide; SSZ: sulfasalazine; HCQ: hydroxychloroquine.

**Table 2:** Associations between baseline serum levels of cytokines and clinical parameters.

	ESR	CRP	HAQ-DI	VAS	DAS28	RF	ACPA
IFN-*γ*	*r* = 0.4	*r* = 0.2	*r* = 0.2	*r* = 0.4	*r* = 0.1	*r* = 0.2	*r* = 0.2
	*p* = 0.0371	*p* = 0.6560	*p* = 0.5965	*p* = 0.8113	*p* = 0.4572	*p* = 0.3761	*p* = 0.2413
IL-6	*r* = 0.1	*r* = 0.2	*r* = 0.2	*r* = 0.1	*r* = 0.09	*r* = 0.08	*r* = 0.09
	*p* = 0.0081	*p* = 0.0019	*p* = 0.0782	*p* = 0.0572	*p* = 0.0389	*p* = 0.6491	*p* = 0.6422
IL-17	*r* = 0.5	*r* = 0.3	*r* = 0.2	*r* = 0.07	*r* = 0.2	*r* = 0.4	*r* = 0.3
	*p* = 0.0812	*p* = 0.1087	*p* = 0.5182	*p* = 0.6899	*p* = 0.3006	*p* = 0.0464	*p* = 0.08443
IL-35	*r* = −0.4	*r* = −0.5	*r* = −0.4	*r* = −0.4	*r* = −0.4	*r* = 0.1	*r* = 0.1
	*p* = 0.0300	*p* = 0.0013	*p* = 0.0202	*p* = 0.0164	*p* = 0.0100	*p* = 0.6721	*p* = 0.6037
TNF-*α*	*r* = 0.4	*r* = 0.02	*r* = 0.1	*r* = 0.1	*r* = 0.3	*r* = 0.1	*r* = 0.3
	*p* = 0.0174	*p* = 0.0074	*p* = 0.0165	*p* = 0.0539	*p* = 0.0098	*p* = 0.5964	*p* = 0.1586

Abbreviations: IL: interleukin; ESR: erythrocyte sedimentation rate; CRP: C-reaction protein; HAQ-DI: Health Assessment Questionnaire-Disability Index; VAS: visual analogue scale; DAS28: disease activity score in 28 joints based on erythrocyte sedimentation rate; RF: rheumatoid factor; ACPAs: anticyclic citrullinated protein antibodies.

**Table 3:** Comparison of responders and nonresponders at week 24 after tofacitinib treatment.

	Responders(*n* = 25)	Nonresponders(*n* = 7)	*p* value
*Demographics*			
Age, median (IQR), years	43 (31-62)	45 (35-59)	0.23
Sex (F/M)	19/6	6/1	0.69
Disease duration, median (IQR), years	5.8 (4.9-14.8)	6.2 (5.3-15.2)	0.55
*Disease characteristics*			
CRP, median (IQR), mg/L	11.8 (9.0-15.6)	22.7 (21.4-66.2)	<0.0001
DAS28-ESR, median (IQR)	2.0 (1.9-2.3)	3.3 (3.2-4.9)	<0.0001
RF positive, *n* (%)	38.5 (20.0-93.7)	53.8 (20-1083)	0.66
ACPA positive, *n* (%)	17 (17-32)	17 (17-231)	0.24
*Medication type*			
Tofacitinib, *n* (%)	25 (100.0)	7 (100.0)	1.0000
NSAID, *n* (%)	20 (80.0)	6 (85.7)	0.49
CS, *n* (%)	5 (20.0)	3 (42.6)	
Oral prednisone dose (mg/day)	10 (5-10)	10 (5-10)	1.000
Concomitant DMARDs			
MTX, *n* (%)	12 (48.0)	3 (42.8)	0.53
LEF, *n* (%)	9 (36.0)	4 (57.1)	0.62
SSZ, *n* (%)	3 (12.0)	1 (14.3)	0.44
HCQ, *n* (%)	18 (72.0)	5 (71.4)	0.27
*Serum cytokines*			
IFN-*γ*	1.8 (1.1-2.2)	5.5 (5.1-6.0)	<0.0001
IL-6	3.5 (2.5-5.8)	11.9 (6.9-12.9)	0.011
IL-17	4.2 (3.4-5.9)	12.5 (5.3-17.3)	0.0048
IL-35	104.3 (72.2-194.5)	28.2 (27.5-29.7)	0.0011
TNF-*α*	2.6 (2.1-3.2)	4.1 (2.6-5.7)	0.0943

Abbreviations: RA: rheumatoid arthritis; IQR: interquartile range; F: femal; M: male; ESR: erythrocyte sedimentation rate; CRP: C-reaction protein; TJC: tender joint count; SJC: swollen joint count; HAQ-DI: Health Assessment Questionnaire-Disability Index; VAS: visual analogue scale; DAS28: disease activity score in 28 joints based on erythrocyte sedimentation rate; RF: rheumatoid factor; ACPAs: anticyclic citrullinated peptide antibodies; NSAID: nonsteroidal anti-inflammatory drug; CS: corticosteroid; MTX: methotrexate; LEF: leflunomide; SSZ: sulfasalazine; HCQ: hydroxychloroquine.

## Data Availability

The Research Article data used to support the findings of this study are available from the corresponding author upon request.
